# Annual Meeting of the Oneida County Veterinary Medical Society

**Published:** 1895-03

**Authors:** F. Morrow

**Affiliations:** Secretary


### ANNUAL MEETING OF THE ONEIDA COUNTY VETERINARY MEDICAL SOCIETY.

The annual meeting of the Oneida County Veterinary Medical Society was called to order by the President, February 19, 1895.

Those answering their names at the roll-call were President Dr. H. G. Hollingworth, Secretary Dr. F. Morrow, Drs. Currie, Huff and Findlay. After the President's address an application for membership of Dr. J. S. Elliot, of Clinton, was read after paying the usual fees. A communication was received from Dr. R. M. Weightman, of Waterville, regretting his inability to be present.

It being the annual meeting election of officers was in order. The old officers were re-elected.

Moved and carried that we hold our meetings quarterly on the first Tuesday in every quarter.

Dr. Hollingworth read a paper on "Tuberculosis."

Moved and carried that a vote of thanks be given the essayist. Dr. Huff then read a very lengthy and interesting paper on "Hygiene," of which consideration was postponed until the next meeting for lack of time. A vote of thanks was accorded the essayist for his valuable paper. The meeting then adjourned to meet again the first Tuesday in May.

F. MORROW, V.S.
Secretary.

